# Psychometric properties of a short form for the Coping Inventory for Stressful Situations (CISS-SF)

**DOI:** 10.1371/journal.pone.0356279

**Published:** 2026-08-17

**Authors:** Amy Van Elswyk, Colin T. Henning, Laura J. Summerfeldt, James D. A. Parker

**Affiliations:** Trent University, Peterborough, Canada; SUNY Empire, UNITED STATES OF AMERICA

## Abstract

The Coping Inventory for Stressful Situations (CISS) is a widely used measure of coping that was developed to assess three basic coping styles: task-oriented, emotion-oriented, and avoidance-oriented coping. The present study examined the factor structure and psychometric properties (internal reliabilities and temporal stability) of a short form for the CISS (CISS-SF) in a large longitudinal sample of community-based adults who completed the coping measure on 3 separate occasions approximately 1 year apart. The 3-factor model composed of 21 coping items provided acceptable fit to the sample data. The model was invariant across gender and age groups. Internal reliabilities for the scales were acceptable across multiple administrations (by gender and age groups), while 1 and 2-year test-retest correlations were also moderate and consistent with what would be expected for stable coping style constructs. Preliminary validity of the CISS-SF was explored with respect to self-report measures of big five personality and depression and anxiety symptoms. The relationships between coping, big five personality, and mental-health outcomes were found to be consistent with previous research on coping. Overall, the results suggest that the CISS-SF is a valid and reliable brief multi-dimensional measure of coping styles.

Compared to work on related constructs like “stress” or “defense,” the coping area is a relatively recent development in the history of psychology [1]. For example, the category for “coping” was not included in *Psychological Abstracts* until 1967 [2]. Despite having been introduced late in the discipline, coping has since proven to be an extremely popular construct. A recurring observation in reviews on the topic, even as the new field was just emerging [3], was the dramatic expansion of research interest on coping [4–7]. Taking stock of the current literature, the coping area would appear to have reached a new milestone with respect to research activity. Using the number of research sources as an indicator, the database in the ISI Web of Knowledge [8] contains 40,931 items with key-terms for “coping” in the period from 1990 to 2025. While there has been steady interest in coping research over the last 35 years, one-third (33%) of this literature has been published within the last five years alone.

Given this volume of research, it is not surprising that hundreds of distinct coping assessment tools have been developed since the 1970s [9–12]. Though the sheer variety of measures poses a challenge for organizing research findings, much of the coping literature is concentrated around a relatively small number of assessment tools [13]. In turn, this smaller set of coping measures can be sorted into two broad approaches: “intraindividual” versus “interindividual” [1,4,14]. The intraindividual approach to coping, exemplified by measures like the *Ways of Coping Questionnaire* (WCQ [15]), attempts to identify basic strategies used by individuals in specific types of stressful situations. The interindividual approach, on the other hand, attempts to identify basic coping styles, or habitual coping strategies, used by individuals across different types of stressful situations. The *Coping Inventory for Stressful Situations* (CISS [16]), for example, is a widely used interindividual coping measure [13]. There are also coping measures, like the COPE [17], the most widely used coping measure in the literature [13], developed to have both intraindividual (situation specific) and interindividual (dispositional) forms.

Although it has long been recognized that individuals have access to a broad range of potential coping strategies and reactions in any given situation [3], coping researchers have generally assumed that these responses can be organized into a relatively small number of basic coping dimensions or strategies. Importantly, these broader dimensions appear to overlap across both interindividual and intraindividual assessment approaches [1]. The number of basic coping dimensions that appear on the most widely use coping measures range from the 2 higher-order coping dimensions initially proposed by Folkman and Lazarus [18] to the 15 facets of coping measured by the COPE [17]. Of these numerous strategies and dimensions, however, three core coping dimensions have emerged to be of particular importance in the literature—namely, task-oriented, emotion-oriented, and avoidance-oriented coping [19,20].

Task-oriented (or problem-oriented) coping involves strategies used to appraise a stressful situation to make it more comprehensible, find solutions to address the source of the stress, or reduce the salience of the stressor on the individual [21,22]. Task-oriented coping is associated with better judgement and decreased anxiety [23,24]. In contrast, emotion-oriented coping involves focusing on the emotional consequences of a stressor rather than the stressor itself, including self-preoccupation and fantasizing reactions [4,24]. Avoidance-oriented coping detaches individuals further by having them seek to avoid stressful situations, including habitually denying or distracting oneself from the stressor [4,22,24].

## Measuring task, emotion and avoidance-oriented coping

Although there are other coping measures that tap comparable constructs, the CISS was explicitly designed as a self-report measure of broad task, emotion, and avoidance-oriented coping constructs [4,16,23]. The initial phase of creating the CISS involved compiling a comprehensive list of items representing diverse coping responses [16]. A preliminary 70-item inventory was administered to 559 undergraduate participants who were asked to gauge their general reactions when faced with challenging, stressful, or upsetting situations. The 70 items underwent several waves of factor analysis to reveal three factors (Task, Emotion, and Avoidance-Oriented coping), with factor analyses conducted separately for men and women yielding almost identical factor structures [16]. Due to discrepancies in the number of items loading on the three subscales, additional items were developed with the aim of achieving subscales with equal item counts. A subsequent 66-item scale was administered by Endler and Parker [16] to two different adult samples. Following similar analyses with the original item-pool, a 48-item scale was derived by eliminating redundant or poorly loading items, subsequently known as the CISS [16]. The final 3-factor model for the CISS, three 16-item scales assessing task-, emotion-, and avoidance-oriented coping, was validated using multiple independent samples [16,23].

## Short form for the CISS

In the research literature there has been a growing trend of abbreviated versions of commonly used assessment tools [25]. This practice is aimed at streamlining the assessment process by enabling efficient evaluation of multiple constructs while reducing the cognitive burden on research participants. Not surprisingly, short forms have been developed for several widely used coping measures [26–28]. In the case of the CISS, a “short form” has been in circulation since 2006 [29], however, this specific form was not originally developed by the authors of the CISS. When the 2^nd^ edition of the CISS manual was developed in the late 1990s [30], the authors identified 21 CISS items (three 7-item scales for task, emotion, and avoidance-oriented coping) that could be used to assess coping behaviours in specific stressful situations (e.g., coping with job loss or coping with marital separation, etc.). The development of a brief CISS-situation specific coping form (CISS-SSC) alongside the original CISS duplicated the approach to having both intraindividual and interindividual forms used by other coping scales like the COPE [17]. Cohan et al. [29] used the CISS-SSC items to operationalize a short version of the original CISS (i.e., a short interindividual coping measure of general coping styles) and examined the psychometric properties of this new form with data from undergraduates and community-based adults. Although the authors found evidence to support the use of this short form for measuring responses to “routine stressors,” they instead chose to evaluate a 4-factor model never used by Endler and Parker in the development of the CISS [4,16,23,30]. The long form of the CISS included two subscales within the Avoidance scale: distraction and social diversion. Building on this structure, Cohan et al. [29] proposed a 4-factor coping model consisting of 7 items for task-oriented coping, 7 items for emotion-oriented coping, 3 items for distraction coping, and 3 items for social diversion coping, while also dropping one of the original avoidance items from the CISS-SSC.

Since its publication, a confusing body of literature has appeared using the CISS item set, and short form scoring developed by Cohan et al. [29], with the psychometric properties of this brief interindividual coping measure being very inconsistent across studies [31–35]. A contributing factor is that much of this research has utilized non-English versions of the item pool without adequate documentation on how these translated item sets were developed, increasing the likelihood of producing scales with problematic psychometric properties (see [36] for a more detailed review of this problem). Additionally, the decision by Cohan et al. [29] to derive 4 subscales from a 20-item pool likely undermined the psychometric properties of the new CISS form from the start, given the inherent challenges associated with 3-item scales (e.g., low reliability, inadequate construct representation [37–39]).

## Present study

To date, no study has revisited Cohan et al.‘s [29] work and systematically evaluated the use of the 21 items from the CISS-SSC as an interindividual measure of general coping styles, particularly in line with the original 3-factor conceptual model of coping (Task, Emotion, and Avoidance-oriented coping) as proposed in the development of the original CISS and consistent with broader theoretical models of coping [4,16,23]. Using data from a large sample of adults who completed a short form of the CISS (called the CISS-SF in the remainder of this document) multiple times over several years [40], the present study sought to examine whether the original 3-factor model used in the development of the CISS [4,16,23] could be replicated in a large community-based sample. The longitudinal nature of the dataset also allowed for an exploration of test-retest reliability across a multi-year period. Using several personality and mental health variables in the dataset, the present study further examined the construct validity of the CISS-SF using variables comparable to Cohan et al. [29]—anxiety, depression, and basic personality.

## Method

### Participants and procedures

The present study used data from 1372 adults (602 men and 770 women) who participated in the Leisure, Lifestyle, and Lifecycle Project (LLLP [40]). The data is publicly available at Greo Evidence Insights and can be accessed at https://www.greo.ca/en/greo-resource/data-repository.aspx. Ethical approval for data collection for the LLLP was originally obtained from the University of Alberta Health Research Ethics Board, the University of Calgary Conjoint Health Research Ethics Board, and the University of Lethbridge Human Subjects Research Committee [41]. Subsequently, ethical approval for secondary analysis of the LLLP data in the present study was obtained from the research ethics board at the authors’ home institution (file no. 25742). All participants provided informed consent for data collected for this study.

Initial recruitment for the LLLP at wave one involved telephone sampling, followed by in-person interviews and the administration of various self-report questionnaires. Participants were recruited from four locations in Alberta, Canada (Edmonton, Calgary, Lethbridge area, and Grand Prairie area) and were also oversampled based on age and gender specific cut-offs for gambling activities, as well as for specific age groups (17- to 20-year-olds; 23- to 25-year-olds; 43- to 45-year-olds and 63- to 65-year-olds). There were four additional waves (each ~1 year apart) where data collection was completed using online surveys, and a small portion (less than 10% of the sample) completed paper and pencil versions.

A total of 1,372 adults 18 years or older participated in wave one of data collection. The CISS-SF was included in waves 2, 3 and 4; data was available for 1145 individuals (478 men and 667 women) at wave 2, 1002 individuals (406 men and 596 women) at wave 3, and 1026 individuals (414 men and 612 women) at wave 4 (gender was missing for a small number of cases). At wave one, participants were on average 37.47 years old (SD = 17.26; range: 18–65). Approximately 89.87% of participants identified as White/Caucasian, 2.41% as Asian, and the remaining 7.72% as belonging to various other ethnic groups. Other demographic features of the sample have been described in detail elsewhere [40,41]. Ethical approval for use of the data in the present study was obtained from the research ethics board at the authors’ home institution (file no. 25742). All participants provided informed consent for data collected for this study.

### Measures

#### Coping.

As already noted, the CISS was originally developed as an interindividual coping measure of basic coping styles [4,16,23]. However, Endler and Parker [30] subsequently created a 21-item scale from the CISS item pool that could be used to measure situation-specific coping (CISS-SSC). The present study used the same 21 items from the CISS-SSC, but with instructions to respondents from the original CISS designed to measure basic coping styles. Unlike the measure developed by Cohan et al. [29], the CISS-SF has 21 items that measure 3 basic coping style dimensions: 7-items for task-oriented coping; 7 items for emotion-oriented coping; and 7-items for avoidance-oriented coping.

#### Mental health.

The Personality Assessment Inventory [42] is a 344-item self-report measure designed to measure various personality and psychopathology traits. The PAI uses a four-point Likert scale format (1 = “false, not at all true” to 4 = “very true”), with 8–24 items across 22 clinical subscales (e.g., anxiety, schizophrenia). The PAI has previously demonstrated good reliability and validity [42].The present study used only the anxiety and depression subscales from the PAI collected at wave 4.

#### Basic personality.

The NEO-FFI is a shortened version of the NEO-PI-R [43] designed to assess the five-factor model of personality (i.e., openness to experience, conscientiousness, extraversion, agreeableness, and neuroticism). There is extensive support for the validity and reliability of the NEO-FFI [43]. The present study used data on the NEO-FFI collected at wave 1 (the only wave in which the personality measure was used).

### Statistical procedure

#### Factor structure.

Confirmatory Factor Analysis (CFA) was used to assess the proposed 3-factor structure of the 21 CISS-SF items, separately for waves 2, 3, and 4. All CFA models were estimated using Diagonally Weighted Least Squares (DWLS) as the estimation method. To assess goodness of fit, we used the following robust indices: Comparative Fit Index (CFI), Root Mean Square Error of Approximation (RMSEA) with its 90% confidence interval (CI), and Standardized Root Mean Square Residual (SRMR). The following graded fit criteria were used based on previously recommended cut-offs to evaluate the quality of each model [44,45]: CFI ≥ 0.95, RMSEA ≤ 0.05, and SRMR ≤ 0.08 for good fit; CFI ≥ 0.90, RMSEA ≤ 0.08, and SRMR ≤ 0.10 for acceptable fit.

#### Measurement invariance.

Measurement invariance of the 3-factor structure for the CISS-SF was evaluated with respect to gender (males and females) and age (age groups for 18–25 and 42–65) using data from wave 2, as well as with respect to time (wave 2, wave 3, and wave 4). We tested for measurement invariance for gender, age and time separately, within a multiple-group CFA framework by fitting the same model for each sample (e.g., males and females) simultaneously and imposing increasingly restrictive cross-group equality constraints on its parameters. We began with a baseline model constraining only the number of factors and the pattern of factor loadings (configural invariance), then conducted a test of invariant factor loadings (metric invariance), and then a test of invariant factor covariances (structural invariance). Invariance was assumed to hold if these constrained models fit the data well and if there was minimal difference in their fit from that of the baseline model [46]. Because of excessive Type I error rates associated with the chi-square difference test in large samples, we adapted a procedure recommended by Vandenberg and Lance [47] and evaluated the relative fit of constrained models using change in CFI, RMSEA and SRMR values, with minor differences of ≤.02 indicating equivalent fit.

#### Incremental validity.

To examine the relationships between coping, the five-factor model of personality, and mental health symptomatology, Pearson product-moment correlations were calculated (separately by gender) between the CISS-SF scales at wave 2, scales for the five-factor model at wave 1, and the scales for depression and anxiety at wave 4. For all Pearson product-moment correlations, missing cases were excluded in a pairwise fashion across the waves of available data. To evaluate incremental validity of the CISS-SF with respect to predicting depression and anxiety symptomatology, two hierarchical multiple regressions were conducted (separately by gender). For each regression model, mental health symptom scale scores were used as the dependent variable and the 5 scales from the NEO-FFI at wave 1 were entered as the predictor variables in step 1, followed by the three CISS-SF scales at wave 2 entered as predictor variables in step 2. Separate regression models were conducted with either depression or anxiety as the dependent variable.

## Results

### Factor structure

CFA was used to assess the 3-factor theoretical model for the CISS-SF items separately for each wave of data. The 3-factor model for the CISS-SF items showed acceptable model fit for all three waves: Wave 2, CFI = .924, SRMR = .076, and RMSEA = .075, 90% CI = [.071,.078]; Wave 3, CFI = .915, SRMR = .085, and RMSEA = .084, 90% CI = [.080,.088]; and Wave 4, CFI = .919, SRMR = .083, and RMSEA = .081, 90% CI = [.077,.085]. For all three waves, item-to-factor standardized parameter estimates were statistically significant, as were associations among each of the latent variables (see Table 1).

**Table 1:** Standardized item-to-factor parameter estimates and inter-factor estimates from CFAs of the three-factor CISS-SF model (separately by wave).

Item	Wave 2	Wave 3	Wave 4
*Task-Oriented*			
1. Focus on the problem…	.662*	.747*	.771*
4. Think about how I…	.765*	.781*	.793*
7. Determine a course…	.829*	.850*	.838*
10. Work to understand…	.810*	.839*	.844*
13. Take corrective action…	.716*	.734*	.729*
16. Think about the event…	.763*	.742*	.765*
19. Analyze the problem…	.658*	.723*	.717*
*Emotion-Oriented*			
2. Blame myself…	.624*	.679*	.674*
5. Feel anxious about…	.716*	.733*	.742*
8. Blame myself for…	.758*	.797*	.788*
11. Become very upset	.703*	.723*	.722*
14. Blame myself for…	.778*	.785*	.761*
17. Wish that I could…	.620*	.647*	.681*
20. Focus on my general…	.742*	.766*	.738*
*Avoidance-Oriented*			
3. Treat myself to…	.619*	.681*	.682*
6. Go out for a…	.638*	.684*	.653*
9. Buy myself something	.610*	.640*	.610*
12. Visit a friend	.635*	.625*	.620*
15. Spend time with…	.579*	.544*	.581*
18. Phone a friend	.670*	.580*	.587*
21. Take time off…	.457*	.454*	.541*
Inter-Factor Estimates			
Task with Emotion	−.164*	−.143*	−.099*
Task with Avoidance	.134*	.076*	.171*
Emotion with Avoidance	.437*	.498*	.471*

Note. N = 1145 for wave 2, 1002 for wave 3 and 1026 for wave 4; * *p* <.05.

### Measurement Invariance across gender, age, and time

For measurement invariance across gender, the configural invariance model showed an adequate fit to the data, CFI = .925, SRMR = .080, and RMSEA = .072, 90% CI = [0.068, 0.076], indicating that the 3-factor model was equivalent for men and women. The metric invariance model was comparably well fitting, CFI = .923, SRMR = .081, and RMSEA = .071, 90% CI = [0.067, 0.075], indicating the CISS-SF items did not function differently for the two groups (change in CFI was.002, SRMR was.001 and in RMSEA was.001). The structure invariance model was comparably well fitting [CFI = .911, SRMR = .086, and RMSEA = .076, 90% CI = [0.073, 0.080], indicating that there were limited gender differences in either the nature of the latent coping dimensions on the CISS-SF or the degree of overlap among them (change in CFI was.014, SRMR was.006 and RMSEA was.004).

For measurement invariance across age groups (age groups for 18–25 and 42–65), the configural invariance model showed an adequate fit to data, CFI = .923, SRMR = .080, RMSEA = .073, 90% CI is [0.070, 0.077], indicating the 3-factor model was equivalent between age groups. The metric invariance model was comparably well fitting, CFI = .919, SRMR = .082, RMSEA = .074, 90% CI = [0.070, 0.077]), indicating the CISS-SF items did not function differently for the two groups (change in SRMR was.002 and in RMSEA was.001). The structure invariance model was adequately fitting (CFI = .898, SRMR = .089, RMSEA = .080 and 90% CI = [0.077, 0.084]), indicating that there were limited age differences in either the nature of the latent coping dimensions on the CISS-SF or the degree of overlap among them (change in SRMR was.009 and in RMSEA was.007).

For measurement invariance across time, the configural invariance model also indicated an adequate fit to the data, CFI = 0.919, SRMR = 0.081, RMSEA = 0.080, 90% CI [0.078, 0.082], indicating the 3-factor model was equivalent across all three time-points. The metric invariance model was also adequately fitting, CFI = 0.919, SRMR = 0.082, RMSEA = 0.078, 90% CI [0.075, 0.080], indicating the CISS-SF items did not function differently across time (change in CFI was.000, SRMR was.001 and RMSEA was.002). The structural invariance model was adequately fitting, CFI = 0.917, SRMR = 0.079, RMSEA = 0.076, 90% CI [0.074, 0.078], indicating that there were limited differences across time in either the nature of the latent coping dimensions on the CISS-SF or the degree of overlap among them (change in CFI was.002, SRMR was.002, and RMSEA was.004).

### Reliability and age/gender differences for CISS-SF scales

Means, standard deviations (SD), and Cronbach’s alphas for the CISS-SF scales (separately by gender and age group) are presented in Table 2. All CISS-SF subscales showed moderate to high internal reliabilities with Cronbach’s alphas ranging from.80 to.92 for the total sample across the 3 waves of data (alphas for men or women, as well as for younger or older adults were also moderate to high in value). Table 3 presents the test-retest correlations conducted for the CISS-SF scales across the 3 waves of data (separately by gender). For test-retest correlations, missing cases were excluded in a pairwise fashion. A consistent pattern of moderate 1-year and 2-year test-retest correlations were found for all CISS-SF scales, ranging from.55 to.65 for the total sample (correlations for men or women, as well as for younger or older adults were relatively consistent in magnitude).

**Table 2:** Means, standard deviations and alpha coefficients by gender and wave for the CISS-SF scales.

Wave	Task-Oriented	Emotion-Oriented	Avoidance-Oriented
	Mean (SD) Alpha	Mean (SD) Alpha	Mean (SD) Alpha
*Wave 2*			
Men (n = 478)	3.81 (0.86).91	2.27 (0.82).86	2.33 (0.84).82
Women (n = 667)	3.81 (0.78).89	2.62 (0.90).88	2.76 (0.78).76
Young (n = 508)	3.76 (0.74).87	2.62 (0.91).88	2.86 (0.77).75
Old (n = 637)	3.85 (0.86).91	2.35 (0.84).86	2.36 (0.81).80
Total (n = 1145)	3.81 (0.81).90	2.47 (0.88).87	2.58 (0.83).80
*Wave 3*			
Men (n = 406)	3.86 (0.86).92	2.06 (0.81).87	2.19 (0.80).81
Women (n = 596)	3.71 (0.82).90	2.45 (0.92).89	2.58 (0.77).77
Young (n = 435)	3.78 (0.81).91	2.40 (0.96).90	2.65 (0.78).77
Old (n = 567)	3.76 (0.86).91	2.21 (0.84).87	2.25 (0.78).79
Total (n = 1002)	3.77 (0.84).91	2.30 (0.84).89	2.42 (0.81).80
*Wave 4*			
Men (n = 414)	3.78 (0.92).93	2.05 (0.78).87	2.19 (0.79).82
Women (n = 612)	3.76 (0.84).90	2.41 (0.89).89	2.64 (0.79).78
Young (n = 447)	3.88 (0.75).89	2.39 (0.89).89	2.70 (0.79).78
Old (n = 579)	3.68 (0.95).93	2.17 (0.83).88	2.27 (0.80).80
Total (n = 1026)	3.77 (0.87).92	2.26 (0.87).89	2.46 (0.82).80

**Table 3:** Test-rest correlations for CISS-SF scales (separately by gender and time-period).

Scale/Time-Period	Total Sample	Males	Females	Young	Old
Task-Oriented					
wave 2 with wave 3	.56*	.53*	.58*	.58*	.55*
wave 2 with wave 4	.55*	.54*	.56*	.52*	.58*
wave 3 with wave 4	.57*	.52*	.60*	.59*	.56*
N	1372	602	770	656	716
Emotion-Oriented					
wave 2 with wave 3	.58*	.56*	.56*	.55*	.59*
wave 2 with wave 4	.59*	.53*	.60*	.58*	.58*
wave 3 with wave 4	.65*	.60*	.65*	.65*	.64*
N	1372	602	770	656	716
Avoidance-Oriented					
wave 2 with wave 3	.56*	.50*	.56*	.49*	.55*
wave 2 with wave 4	.59*	.58*	.54*	.50*	.59*
wave 3 with wave 4	.59*	.53*	.59*	.57*	.56*
N	1372	602	770	656	716

Note. * *p* < .05.

Gender differences on the various CISS-SF scales were evaluated using separate independent *t*-tests. For wave 2, women scored significantly higher on the emotion-oriented scale [*t* (1143) = 6.75, p < .001, *d* = 0.41], and the avoidance-oriented scale [*t* (1143) = 8.80, p < .001, *d* = 0.53]. For wave 3, men scored significan*t*ly higher on the task-oriented scale [*t*(1000) = −2.82, p < .001, *d* = 0.18]; women scored higher on both *t*he emotion-oriented scale [*t*(1000) = 6.97, p < .001, *d* = 0.45], and avoidance-oriented scale [*t*(1000) = 7.82, p < .001, *d* = 0.50]. For wave 4, women scored higher on *t*he emotion-oriented scale [*t* (1024) = 6.53, p < .001, *d* = 0.43], and the avoidance-oriented scale [*t* (1024) = 9.04, p < .001, *d* = 0.57]. Overall, gender differences across the CISS-SF scales were generally modes*t* in magnitude.

Age differences on the various CISS-SF scales were evaluated using separate independent *t*-tests. For wave 2, the younger group scored significantly higher on the emotion-oriented scale [*t* (1143) = 5.31, *p* < .001, *d* = 0.31] and the avoidance-oriented scale [*t* (1143) = 10.56, *p* < .001, *d* = 0.63]. For wave 3, *t*he younger group again scored significantly higher on both the emotion-oriented scale [*t*(1000) = 3.35, *p* < .001, *d* = 0.21], and the avoidance-orien*t*ed scale [*t*(1000) = 7.96, *p* < .001, *d* = 0.51], than the older group. For wave 4, *t*he younger group scored significantly higher on the task-oriented scale [*t*(1024) = 3.64, *p* < .001, *d* = 0.23], the emotion-oriented scale [*t*(1000) = 4.00, *p* < .001, *d* = 0.26], and the avoidance-oriented scale [*t*(1000) = 8.73, *p* < .001, *d* = 0.54], than *t*he older group. Overall, age differences across the CISS-SF scales were generally modest in magnitude.

### Incremental validity of the CISS-SF predicting mental health symptomatology

Table 4 presents the means and standard deviations for all coping, personality, and mental health variables (separately by gender), as well as correlations among all variables. Correlations between the CISS-SF scales and both depression and anxiety symptoms were generally significant but small to moderate in magnitude (ranging from.21 to.44 for males and.24 to.52 for females) with the exception of the avoidance-oriented coping scale which was not significantly associated with depression symptoms. Additionally, correlations between the CISS-SF scales and the big five personality scales were generally significant but small in magnitude (ranging from.08 to.29 for males and.05 to.32 for females), with the exception of moderate to strong correlations between emotion-oriented coping and neuroticism for both males and females. The overall pattern of correlations between the big fiver personality scales and the CISS-SF-scales showed neuroticism was negatively correlated with task-oriented coping, extraversion was negatively correlated with emotion-oriented coping, and both agreeableness and conscientiousness were negatively correlated with both emotion-oriented and avoidance-oriented coping. In contrast, openness had very weak correlations across all three coping scales.

**Table 4:** Correlations for NEO-FFI scales, CISS-SF scales, anxiety, and depression (separately by gender).

Measure	1	2	3	4	5	6	7	8	9	10	Mean	SD
1 Depress	—	0.72*	−0.24*	0.38*	0.07	0.47*	−0.35*	−0.15*	−0.10*	−0.25*	14.21	9.75
2 Anxiety	0.78*	—	−0.28*	0.52*	0.25*	0.58*	−0.21*	−0.20*	−0.06	−0.24*	15.23	10.35
3 Task	−0.22*	−0.21*	—	−0.22*	0.07	−0.27*	0.14*	0.13*	0.20*	0.32*	3.81	0.78
4 Emotion	0.42*	0.44*	−0.05	—	0.31*	0.50*	−0.14*	−0.24*	−0.05	−0.26*	2.62	0.90
5 Avoid	0.06	0.21*	0.16*	0.39*	—	0.21*	0.11*	−0.16*	0.16*	−0.15*	2.76	0.78
6 Neurot.	0.51*	0.55*	−0.27*	0.48*	0.22*	—	−0.35*	−0.42*	−0.04	−0.49*	46.98	10.96
7 Extra.	−0.35*	−0.24*	0.14*	−0.20*	0.17*	−0.41*	—	0.26*	0.21*	0.23*	52.49	10.76
8 Agree.	−0.29*	−0.24*	0.09*	−0.16*	−0.14*	−0.41*	0.22*	—	0.04	0.36*	51.38	11.56
9 Open.	−0.06	0.01	0.19*	0.08	0.18*	0.03	0.17*	−0.04	—	−0.03	56.08	10.16
10 Cons.	−0.29*	−0.26*	0.15*	−0.29*	−0.20*	−0.49*	0.28*	0.27*	−0.16*	—	48.46	11.09
Mean	13.69	11.72	3.81	2.27	2.33	48.06	53.01	49.51	55.22	47.59		
SD	9.52	8.65	0.86	0.82	0.84	11.06	11.31	11.30	11.22	10.77		

Values for men below the diagonal; Depress = total depression at wave 4, Anxiety = total anxiety at wave 4, Task = task-oriented coping at wave 2, Emotion = emotion-oriented coping at wave 2, Avoid = avoidance-oriented coping at wave 2, Neurot = neuroticism at wave 1, Extra = extraversion at wave 1, Agree = agreeableness at wave 1, Open = openness at wave 1, Cons = conscientiousness at wave 1. N for men is 602 and N for women is 770; * *p* < .05.

To examine the unique contribution of coping to the prediction of mental health symptoms, while controlling for big five personality, a series of hierarchical multiple regression analyses were conducted (separately by gender), with depression and anxiety symptoms as dependent variables. For depression, the predictors accounted for 30% of the variance for women and 35% of the variance for men (see Table 5). For women, the CISS-SF scales accounted for an additional 4% of variability in depression above big five personality, whereas for men the CISS-SF accounted for an additional 6%. For women, neuroticism, extraversion, and agreeableness were all unique predictors, whereas for men only neuroticism and extraversion were significant predictors. In terms of the CISS-SF, emotion-oriented coping was the strongest predictor for both women and men. For anxiety, predictors accounted for 42% of the variance in women and 35% of the variance in men (see Table 6). For women, the CISS-SF scales accounted for an additional 9% of the variability in anxiety above personality, whereas for men the CISS-SF accounted for an additional 6% of the variability. Results showed neuroticism was the strongest unique predictor, with all three CISS-SF factors uniquely contributing for both men and women.

**Table 5:** Results of hierarchical multiple regressions for total depression at wave 4.

Variable	*b*	*Beta*	Partial Corr	*R* ^2^
Women (N = 613)				
[Step 1] Neuroticism	0.36*	0.41	0.36	
Extraversion	−0.19*	−0.21	−0.21	
Openness	−0.04	−0.04	−0.05	
Agreeableness	0.08*	0.10	0.10	
Conscientiousness	−0.04	−0.04	−0.04	0.26*
[Step 2] Task	−1.07*	−0.09	−0.09	
Emotion	2.22*	0.21	0.20	
Avoidance	−0.38	−0.03	−0.03	0.30*
Men (N = 415)				
[Step 1] Neuroticism	0.34*	0.38	0.34	
Extraversion	−0.14*	−0.16	−0.16	
Openness	−0.03	−0.04	−0.04	
Agreeableness	−0.06	−0.07	−0.08	
Conscientiousness	−0.05	−0.06	−0.06	0.29*
[Step 2] Task	−0.95	−0.09	−0.10	
Emotion	3.12*	0.28	0.27	
Avoidance	−1.07	−0.10	−0.10	0.35*

Note. * *p* < .05.

**Table 6:** Results of hierarchical multiple regressions for total anxiety at wave 4.

Variable	*b*	*Beta*	Partial Corr	*R* ^*2*^
Women (N = 613)				
[Step 1] Neuroticism	0.58*	0.60	0.52	
Extraversion	0.00	0.00	0.00	
Openness	−0.03	−0.03	−0.04	
Agreeableness	0.03	0.04	0.04	
Conscientiousness	0.04	0.04	0.04	0.33*
[Step 2] Task	−1.80*	−0.14	−0.16	
Emotion	3.12*	0.27	0.28	
Avoidance	1.27*	0.10	0.11	0.42*
Men (N = 415)				
[Step 1] Neuroticism	0.42*	0.51	0.43	
Extraversion	−0.01	−0.02	−0.02	
Openness	0.01	0.01	0.01	
Agreeableness	−0.02	−0.03	−0.03	
Conscientiousness	−0.01	−0.01	−0.01	0.29*
[Step 2] Task	−1.12*	−0.12	−0.13	
Emotion	2.29*	0.22	0.22	
Avoidance	0.59*	0.06	0.06	0.35*

Note. * p < .05.

## Discussion

The present study sought to examine whether the 3-factor model used to guide development of the CISS [4,16,23] would replicate in a shortened version of the scale (CISS-SF) within a large community-based sample. We used a novel set of longitudinal data [40], where participants completed the CISS-SF on three separate occasions (approximately 1 year apart). Confirmatory factor analysis (CFA) results with the multiple “waves” of data demonstrated acceptable fit for the 3-factor model: three 7-item scales assessing task, emotion, and avoidance-oriented coping. Additionally, measurement invariance of the CISS-SF demonstrated that the model held comparably well for both men and women, younger and older adults (17- to 25-year-olds versus 42- to 65-year-olds), indicating that individuals responded to the CISS-SF items in a similar fashion regardless of gender and age. The creation of short forms from a longer assessment tool often comes with reduced internal reliability [25]. However, the alpha coefficients in the current study ranged from.76 to.90 for women,.81 to.93 for men,.75 to.91 for young adults, and.79 to.93 for older adults, suggesting that the short form for the CISS has acceptable internal reliability [48].

The pattern of associations among the three latent coping variables (task, emotion, and avoidance), as well as correlations among the matching scale scores, were indicative of three relatively orthogonal coping dimensions, consistent with previous findings with the normative data for the long form for the CISS [16,30]. It is interesting to note that the relationships between the task and emotion dimensions were consistently negative and very small; the association between these 2 latent variables ranged from −.11 and −.18 across the 3 waves of data. As long emphasized by coping theorists like Folkman and Lazarus [18,49], task (or problem) and emotion-oriented coping are quite distinct responses to stressful situations: “The function of problem-focused coping is to change the troubled person-environment relationship by acting on the environment or oneself. The function of emotion-focused coping is to change either a) the way the stressful relationship with the environment is attended to (a)s in vigilance or avoidance or b) the relational meaning of what is happening, which mitigates the stress even though the actual conditions of the relationship have not changed” [14], p. 238. Thus, the minimal correlation observed between the task-oriented and emotion-oriented coping scales on the CISS-SF is as expected. This pattern of results is also consistent with the results reported for the long form CISS [23,50,51].

The unique nature of the Alberta LLLP dataset [40] also allowed for test-retest correlations of the CISS-SF scales to be examined across 1- and 2-year time intervals. The temporal stability of coping behaviors has long been a point of debate. Early coping theorists like Folkman and Lazarus consistently dismissed the importance of trait measures of coping style [14]. As noted by Folkman [52], “Trait measures of coping are based on the assumption that people are behaviourally consistent across all situations. However, substantial consistency has seldom been found by personality research” (p. 103). Nevertheless, there has long been an interest in the coping area in developing tools for measuring stable basic coping styles [1,53]. When the CISS was originally developed, Endler and Parker [4,16] explicitly proposed that the new measure assessed three “habitual coping strategies” (or styles) used by individuals across stressful situations. Thus, the CISS-SF scales should correlate at least moderately across time. The present study found the scales to be quite stable across time, with 1-year test-retest correlations ranged from.50 to.65 and two-year test-retest correlations similarly ranging from.50 to.59 (see Table 3). These results are higher than the 1-year test-retest correlations from a recent study on basic personality [54]. Moreover, measurement invariance testing showed the CISS-SF items are fully invariant across time with respect to the pattern of factor loadings, the magnitude of factor loadings, and the nature of (and overlap between) coping dimensions, suggesting the coping styles assessed by the CISS-SF are factorially stable across time. Not only are these findings additional evidence that the CISS-SF is a reliable psychometric tool, but the results also support Endler and Parker’s [4,16] contention that coping styles are stable trait constructs.

With respect to the gender differences on the CISS-SF scales, previous research has consistently found gender differences in coping behaviours [55,56]. Women, for example, tend to report using more emotion-oriented coping than men; the opposite pattern of results has also been reported for task-oriented coping [57]. Across the various waves of data, the present study found gender differences in coping scores consistent with previous research using both the long and short forms of the CISS [16,30]. However, consistent with the general coping literature [58] (see also a meta-analysis [59]), the magnitudes of these gender differences were generally quite small.

Potential age-related differences in the use of coping strategies have long been of interest in the coping field [60]. In the small set of relevant studies [61], collective results have been very inconsistent, largely due to the lack of longitudinal designs, as well as important methodological differences in the ways coping has been operationalized (e.g., interindividual vs. intraindividual coping measures). While some researchers have reported individuals shift from using problem-focused coping toward more emotion-focused coping as they age [62–64], others have found either weak or non-existent links between different coping strategies and age [65,66]. In the current study, most of the age-related differences on the CISS-SF were non-significant or quite weak. The one exception was more moderate differences in avoidance-oriented coping across the various waves, with young adults reporting higher scores on this scale than older adults. This is consistent with reports that the use of avoidance-oriented coping decreases in older adulthood [67–69].

### Validity of the CISS-SF

The present study also sought to examine the validity of the CISS-SF by exploring relationships among the coping scales, big five personality, and mental health symptoms. Potential links between coping and big five personality have been of longstanding interest in the coping area [70], with some researchers questioning the need for specific coping assessment tools if coping is nothing more than “personality under stress” [71,72]. At minimum, coping style measures like the CISS-SF are expected to be empirically related to measures of personality traits, but also need to demonstrate incremental validity with respect to predicting important outcomes like health and wellness [23].

In terms of the relationship between the CISS-SF and big five personality, the results obtained for neuroticism (moderate positive association with emotion-oriented coping and negative association with task-oriented coping) closely match previous results obtained using the long form of the CISS [16,30,73]. These findings are also consistent with results for other coping measures and related constructs [70,74]. However, the other personality scales generally correlated very weakly with the CISS-SF scales (with mean absolute *r* values of.17 for both men and women).

Previous research has found that specific coping dimensions assessed by the CISS-SF are linked with a variety of mental health outcomes [22,35]. Cohan et al. [29], in their work to develop a potential short form for the CISS, focused on using the scale to predict depression and anxiety symptomatology. In the present study, depression was found to be moderately positively related to emotion-oriented coping and modestly negatively related to task-oriented coping, measured two years earlier, for both men and women. Previous researchers have similarly found that higher levels of task-oriented coping are linked to lower levels of depressive symptoms, while higher levels of emotion-oriented coping have been linked to higher levels of depressive symptoms [75–77]. Likewise, previous research has found higher levels of task-oriented coping to be linked to lower levels of anxiety symptoms, with the opposite pattern of results found for both emotion-oriented and avoidance coping [22,78]. This pattern of results for anxiety was replicated in the current study (for both men and women).

To explore the incremental validity of the CISS-SF with respect to big five personality, hierarchical multiple regressions were conducted (separately by gender) to determine if the scales predict depression and anxiety symptomology beyond the variability accounted for by big five personality. This is particularly relevant as the constructs connected to the big five routinely predict moderate amounts of depression and anxiety symptomatology [79,80]. For depression symptoms, the results indicated that for both women and men, neuroticism and extraversion were the greatest unique contributors in terms of personality, whereas emotion-oriented coping was the greatest unique contributor in terms of the CISS-SF. Incremental validity was also demonstrated for the CISS-SF, as the coping scales explained an additional 4% of the variability in depression symptoms in women and 6% in men above big five personality. For anxiety symptoms, the results for both women and men indicate that neuroticism was the strongest contributor in terms of personality, whereas all three CISS-SF scales were significant predictors, with task-oriented and emotion-oriented being the strongest. Further incremental validity was demonstrated for the CISS-SF, as the coping scales explained an additional 9% of the variability in anxiety symptoms in women and 6% in men above big five personality.

### Limitations and future directions

Despite these findings, the dataset used in the present study had several limitations that should be noted. El-Gueblay et al. [40] have suggested that participants in earlier waves of the LLLP project may have sought treatment for mental health problems and withdrawn from the study. This potential confound could have influenced the findings of the present study with respect to relationships among the coping, personality, and mental health variables. Furthermore, the results obtained from this study are limited to four regions in Alberta, Canada. Thus, future studies should attempt to replicate our findings using data from other regions within Canada and other countries. Consistent with Cohan et al. [29], the present study also focused on examining whether the CISS-SF scale incrementally predicts only internalizing mental symptomatology (i.e., anxiety and depression) and using self-report data. Thus, future research will be needed to examine whether the CISS-SF scales also predict externalizing mental health symptomatology in theoretically expected and meaningful ways, ideally using multi-method data. Nonetheless, the CISS-SF measures three very distinct coping dimensions that have emerged as particularly significant in the coping field, specifically task-, emotion-, and avoidance-oriented coping [1,19]. Short forms like the CISS-SF are appealing because they can be used in research settings where there are limited time and space for lengthy assessment tools, such as in prospective longitudinal research designs [5,14,68,81].
